# The grass is always greener: a critical look at the lessons and challenges of acupuncture education in America

**DOI:** 10.3389/fmed.2025.1556346

**Published:** 2025-04-22

**Authors:** Zhitao Hou, Zhongren Sun

**Affiliations:** ^1^Heilongjiang University of Chinese Medicine, Harbin, Heilongjiang, China; ^2^Department of Systems Pharmacology and Translational Therapeutics, Perelman School of Medicine, University of Pennsylvania, Philadelphia, PA, United States; ^3^China Association of Acupuncture and Moxibustion, Beijing, China

**Keywords:** acupuncture education, educational reform, traditional Chinese medicine, cultural integration, structuralist and constructivist theories

## Abstract

Acupuncture education forms the foundation for the growth and dissemination of acupuncture in the United States, reflecting its journey toward integration and diversity. Over the past half-century, since the “acupuncture boom,” the U.S. has developed one of the most comprehensive and large-scale acupuncture education systems outside of China. Among these, the diverse schools of acupuncture exemplify the pluralistic evolution of this field in America. This study reviews the formation, transmission, and establishment of various acupuncture schools in the U.S., analyzing their development through the lenses of constructivist and structuralist teaching theories. It identifies key challenges, including fragmented academic frameworks, insufficient theoretical grounding, and a lack of cultural integration. To address these issues, the paper proposes strategies for educational reform, curriculum design, textbook development, and cultural exchange, offering insights to guide the global expansion of acupuncture education and Traditional Chinese Medicine.

## 1 Introduction

Acupuncture education serves as the cornerstone for the dissemination and development of acupuncture in the United States, mirroring its evolution into a flourishing and diverse discipline (1). Since the acupuncture boom in the 1970s, the U.S. has progressively built one of the most comprehensive and large-scale acupuncture education systems outside of China. Jishun Hao, President of American Neuro-Acupuncture Institute and former Chairman of the National Certification Commission for Acupuncture and Oriental Medicine (NCCAOM), has noted that over the past half-century, the U.S. has established a distinctive, diversified acupuncture education system (2–4). As a prominent component of Oriental Medicine, acupuncture has become a pioneer in the globalization of TCM, with its dissemination forming a unique cultural phenomenon (2). During this process, various acupuncture schools have emerged, shaped by the influence of local cultures worldwide.

The term “School,” as used in this paper, primarily refers to a distinctive academic, artistic, or ideological tradition within a specific field (5, 6). However, colloquially, “acupuncture school” also commonly denotes a specific educational institution. To avoid confusion, this manuscript uses “acupuncture schools” when referring to different ideological or clinical traditions, whereas refer specifically to educational establishments providing formal acupuncture training. Acupuncture schools are academic factions that arose during different historical periods or within distinct regional contexts. These schools are defined by shared academic views, knowledge paradigms, or clinical practices and feature clear academic inheritance, unique theoretical perspectives, and notable historical and academic significance (7). The term “School” also conveys a sense of dynamic transmission and development, reflecting the fluid and evolving nature of acupuncture scholarship. In the U.S., acupuncture schools have gradually divided into two contrasting factions: (1) schools rooted in traditional TCM theories such as yin-yang, five elements, and zang-fu organs, represented by Chinese, Japanese, and Korean acupuncture systems; and (2) schools based on modern biological sciences, including anatomy, physiology, and pathology, with medical acupuncture and physical medicine acupuncture being the most prominent examples (1).

Currently, studies on U.S. acupuncture schools are scattered across literature related to education, research, and clinical practice. Comprehensive discussions on acupuncture school education remain scarce. Fan et al. (8). briefly introduced major U.S. acupuncture colleges and summarized the theoretical and clinical practices of specific acupuncture schools. While research on acupuncture’s cross-cultural dissemination is gaining attention, it is primarily led by Chinese scholars, with local practitioners in the U.S. focusing more on scientific research and clinical applications. The systematic study of U.S. acupuncture school education remains underdeveloped, highlighting significant potential for further exploration. This study analyzes acupuncture education through the lenses of structuralist theory, which emphasizes systematic organization and coherent curriculum design, and constructivist theory, which promotes active learner engagement, contextual understanding, and interactive learning processes.

## 2 History and development of acupuncture schools in the United States

### 2.1 Formation of acupuncture schools in the U.S.

The exchange of medical knowledge between nations has a long history. Acupuncture, after being localized in various Eurasian countries, underwent distinct transformations and gave rise to several unique schools of practice. It officially entered the United States during the “acupuncture boom” of the 1970s. Notable examples include the European Five-Element Constitutional Acupuncture, Medical Acupuncture, Physical Medicine Acupuncture, and schools originating from Asian countries like Japan and Korea.

Acupuncture was introduced to Japan and Korea from China as early as the 6th century. Among the acupuncture schools prevalent in the U.S., those based on traditional Chinese medicine (TCM) theories were established earlier. Korea’s Saam Acupuncture, representative of Korean medicine, developed the Four-Needle Method based on Saam acupuncture theory and Korea’s Sasang Constitution Medicine. Rooted in TCM’s Five Elements and meridian theories, it expanded the application of the Five Elements concept to the Five Shu Points, forming a unique constitutional medical framework. Japanese acupuncture, on the other hand, evolved into a coexistence of “classical” and “scientific” schools after the Meiji Restoration, characterized by adaptation, integration, and scientific approaches (9–11). Japanese schools often emphasize abdominal diagnosis and excel in addressing imbalances in body structure and constitution, providing effective treatments for deficiencies in zang-fu organs (11). Similar to Chinese acupuncture, acupuncture practices from Japan and Korea first entered Europe before gaining traction in the United States during the acupuncture boom of the 1970s (12, 13).

Acupuncture began attracting attention in Europe in the mid-17th century, although its relatively late introduction delayed the development of indigenous Western schools (14, 15). These schools often incorporated modern medical theories. In the 1960s, British physiotherapist and homeopath Worsley founded the Five-Element Constitutional Acupuncture School. Rooted in theories from Huangdi Neijing and Nan Jing, it integrated modern psychological diagnostic techniques, achieving remarkable results in treating both mental and physical illnesses. In 1970, Worsley introduced this school of thought to the United States during a lecture tour (15).

In 1952, Travell published the paper The Origins of Myofascial Pain Syndromes, which systematically outlined trigger point theory, marking the official establishment of trigger point therapy (16). While not universally recognized as acupuncture by all practitioners, Travell’s trigger point theory significantly influenced Western acupuncture practices, particularly in medical acupuncture, by integrating anatomical and physiological concepts with traditional acupuncture points and methods. This approach, grounded in anatomy, physiology, pathology, and evidence-based medicine, represents the localization of acupuncture in the West, blending traditional TCM principles with modern medical advancements (16). While medical acupuncture initially did not gain significant attention in the U.S., it achieved a breakthrough following the acupuncture boom of the 1970s, spreading across North America, including Canada (17).

In the 1980s, Mark D. Seem (18) built upon medical acupuncture theory, integrating techniques and practices from various schools in China, Japan, France, and Vietnam (1). By combining meridian theory with modern physical therapy, he founded the Physical Medicine Acupuncture School, also known as New American Acupuncture. This school emphasizes the standardization, proceduralization, and regulation of modern medical practices. It excels in treating conditions such as chronic fatigue syndrome and allergies, aiming to relieve physical and mental tension in patients.

### 2.2 The development of acupuncture schools education in the U.S.

The varied origins of acupuncture, combined with the United States’ open and inclusive cultural ethos, have enabled the growth of numerous acupuncture traditions, resulting in a vibrant and diverse landscape of practices. The foundational framework for acupuncture school education in the U.S. began emerging in the early 1970s.

In 1972, Manaka published An Introduction to Acupuncture, which became instrumental in integrating Japanese acupuncture into the U.S. educational system (19). As of now, 21 institutions in the U.S. provide courses on Japanese acupuncture, with 7 institutions requiring them as part of their core curriculum, particularly in the eastern states (20, 21). Korean acupuncture, exemplified by the Four-Needle Method, is mainly taught in 11 institutions located in the Midwest, with one offering it as a continuing education program (22–24). Schools that do not offer formal courses in Japanese or Korean acupuncture often conduct workshops or seminars, highlighting the foundational presence of these traditions in the U.S. education system.

In 1974, Duggan and Connelly founded the Traditional Acupuncture Institute in Maryland, now known as the Maryland University of Integrative Health (Tai Sophia Institute), which provided a platform for Professor Worsley to introduce the Five-Element Constitutional Acupuncture system in the U.S. (25). Worsley’s books, such as A Guide to Acupuncture and The Acupuncturist’s Handbook, became pivotal in the early stages of this tradition’s education. Currently, 18 institutions offer courses on the Five-Element system, with 12 including it as a required subject.

Medical acupuncture, rooted in Western biomedical science, is predominantly practiced by licensed physicians in the U.S. In 1987, the American Academy of Medical Acupuncture (AAM) was established, becoming the first professional association for medical acupuncturists in North America. The AAM publishes the Medical Acupuncture Journal, provides training and lectures, and focuses on the scientific mechanisms and applications of acupuncture, with minimal reference to traditional acupuncture theories (26).

The Physical Medicine Acupuncture school, a uniquely American development, emphasized education from its inception. In 1979, its founder, Seem, established the Tri-State College of Acupuncture in New York. His works, such as Bodymind Energetics: Toward a Dynamic Model of Health and The Imaging of Acupuncture: Mapping the Body’s Energy Pathways, were used as primary teaching materials, helping the school gain prominence in the eastern U.S. (18, 27–29) (Table 1).

**Table 1:** Characteristics and development of major acupuncture schools in the United States.

Category	Formation period	Theoretical basis	Distinctive theories	Distinctive techniques	Retical features	Representative acupuncture colleges	Number of schools offering courses
Japanese acupuncture	Japanese Meiji Era (1868–1912)	Yin-Yang, Five Elements, and Traditional Chinese Medicine Theories	Yin-Yang Meridian Balance Theory	Six-Division Pulse Diagnosis, Abdominal Diagnosis; Superficial Needling Technique	Focuses on light organ system, heavy meridians; emphasizes root cause treatment, interactive diagnosis and therapy	New England School of Acupuncture; Oriental Medicine and Acupuncture College	21
Korean Sa-Am acupuncture	Korean Joseon Dynasty (1392–1910)		Sa-Am Acupuncture Theory, Four-Element Constitution Theory	Sa-Am Four Needle Method, Hand-Foot Acupuncture	Differentiation and treatment based on phenomena, emphasis on tonification and reduction, integration of acupuncture and moxibustion, emphasis on “De Qi”	International University of Traditional Chinese Medicine	11
European five-element acupuncture	1960s	Modern Biological Theories (Anatomy, Physiology, Pathology)	Five-Element Constitution Theory; Mind–Body-Spirit Balance Theory	Modern Psychological Counseling Methods	Five-Element differentiation and treatment, pulse-based needling, emphasis on regulating the mind, treating root and symptoms separately	Maryland University of Integrative Health	18
Medical acupuncture	1950s		Trigger Point Theory	Manual Stimulation, Dry Needling, Point Injection	Neglects Traditional Chinese Medicine (TCM) theory; uses areas instead of points, focuses on local treatment	American Academy of Medical Acupuncture	0
Physical medicine acupuncture	1980s		Wei-Qi-Ying Treatment Theory	Palpation; Gentle Therapy (Minimal and Superficial Needling)	Combines Eastern and Western methods; emphasizes standardization, proceduralization, and regulation	Tri-State College of Acupuncture	5

## 3 Issues in the development of acupuncture education in the United States

### 3.1 Loose curriculum design and low overall academic standards

Oriental medicine and acupuncture colleges in the U.S. are typically registered by social groups or individuals, most of which are private institutions, leading to significant differences in school size, with enrollment ranging from 30 to 500 students (30, 31). Chinese medicine education institutions in the U.S. are categorized into four main types (32, 33): ① Stand-alone Chinese medicine colleges providing specialized acupuncture training; ② Departments of Chinese medicine in Western medical schools focusing on acupuncture; ③ Continuing education for Western medical personnel; ④ Postdoctoral Chinese medicine programs supported by the NIH. Some profit-driven institutions have led to considerable disparities in the academic levels, faculty quality, and teaching standards of acupuncture education in the U.S. The U.S. established three main bodies to oversee and certify Chinese medicine and acupuncture institutions: the Council of Colleges of Acupuncture and Oriental Medicine (CCAOM), the Accreditation Commission for Acupuncture and Oriental Medicine (ACAOM), and the National Certification Commission for Acupuncture and Oriental Medicine (NCCAOM). The CCAOM handles curriculum design, the ACAOM evaluates educational standards, and the NCCAOM manages certification exams. These organizations have helped legitimize acupuncture institutions and offered guidance and oversight for their operations. As of 2018, 57 institutions had joined the CCAOM, and 62 were accredited by the ACAOM. However, ACAOM requirements for master’s programs consist merely of 2,000 to 3,000 h of study and at least 660 h of clinical practice, without a clear framework for building an acupuncture education system, covering certification, evaluation, or textbook preparation. Because institutions have significant freedom in terms of size, educational level, and training objectives, the framework for acupuncture education in the U.S. lacks systematization and stability, making it difficult to ensure teaching quality (1, 30, 34, 35).

### 3.2 Low exam difficulty and limited inclusion of school styles

The NCCA certification exam is considered a key benchmark for assessing the educational level of acupuncture schools, with 98% of states adopting its content as a standard for acupuncture education and clinical practice (36). The exam includes a theory component and a practical component on point location, where the theory covers fundamental TCM concepts, acupuncture knowledge, and biomedicine, and the practical tests specific point location and sterilized needle techniques. Notably, biomedicine accounts for more than 30% of the content, reflecting a “Westernization” trend in acupuncture education. Despite the exam having a first-time pass rate of over 80%, its difficulty level is comparatively low, leading students to prioritize exam success over clinical skill mastery (15, 37–40). The limitations of the exam content have resulted in insufficient emphasis on school-style curricula in colleges, with acupuncture style-related content gradually diminishing and only offered as special courses in some schools.

### 3.3 Barriers caused by pragmatic education philosophy and professionalization objectives

Under the influence of pragmatic education principles, U.S. acupuncture education focuses more on practical skills training, with relatively less emphasis on theoretical instruction. Most acupuncture school instructors come from clinical practice and tend to emphasize personal experience sharing in their teaching, resulting in a lack of systematic instruction. About two-thirds of the course time is dedicated to acupuncture techniques, with less focus on classical theory studies. Moreover, students’ clinical internships are mostly limited to outpatient cases, lacking in-depth observation of disease progression, which results in graduates being unable to analyze and solve complex cases effectively (2, 38, 39). Some institutions prioritize short-term technical training for economic reasons, neglecting the teaching of classical TCM content. This educational model has hindered the development of acupuncture research, with a severe lack of experimental courses and laboratory studies, obstructing efforts toward acupuncture modernization and interdisciplinary integration.

### 3.4 Disparities in medical theories and cultural frameworks between east and west

In the process of spreading TCM acupuncture in the U.S., conceptual misunderstandings have arisen (10). The U.S. acupuncture community generally believes that TCM acupuncture overemphasizes Zang-Fu theory while neglecting meridians and classic studies. Since TCM practitioners in the U.S. cannot prescribe Western medicine, most acupuncture colleges offer classical TCM courses to explore effective treatment methods through classical theories. However, for English-speaking students, classical courses are challenging to learn, often resulting in a disconnect between theory and clinical practice (1). TCM consists of “Yi Dao” (medical philosophy) and “Yi Shu” (medical techniques). While “Yi Shu” is more adaptable to international standards, “Yi Dao” embodies traditional Chinese philosophy and medical wisdom (18, 28). Such distinctions are especially pronounced in the natural, life, and diagnostic perspectives of Chinese and Western medicine. However, the U.S. acupuncture education system has failed to fully reflect the essence of TCM culture, limiting its clinical application mostly to pain relief. The inadequacy of theoretical education hinders the integration of acupuncture styles into the broader mainstream acupuncture education framework. From a structuralist viewpoint, the fragmented curriculum design and lack of standardized competencies in acupuncture colleges indicate structural weaknesses. Constructivism highlights that neglecting classical theories and interactive educational methods inhibits students’ deeper comprehension and practical application skills.

## 4 Strategies for the U.S. acupuncture education system

### 4.1 Strengthen oversight and reform the teaching system

Structuralist theory suggests that educational effectiveness depends significantly on clearly defined discipline structures, systematic curriculum design, and coherent alignment of educational objectives and competencies (41, 42). Structuralist pedagogy emphasizes the importance of curriculum frameworks that explicitly outline learning sequences, essential competencies, and assessment methods, ensuring systematic and cohesive educational outcomes. The syllabus, as an embodiment of discipline structure, serves as a guide for course organization, teaching content, and progress planning (1). However, due to differences in training goals and institutional characteristics among U.S. acupuncture colleges, as well as restrictions imposed by antitrust laws, a unified syllabus has not yet been established (31). The ACAOM outlines only fundamental requirements for academic structure and hours, failing to offer a detailed framework for curriculum and content, which leaves acupuncture style education without systematic planning. To address this issue, academic organizations led by ACAOM should further improve standards for evaluating teaching quality. Structuralism emphasizes the importance of evaluation feedback in the educational process; thus, it is necessary to enhance oversight and establish a robust evaluation and feedback system. Efforts should encompass hours for core TCM acupuncture and foundational courses, alongside establishing a credit certification system to delineate time allocations between theoretical instruction and hands-on practice. Using U.S. professional standards as a reference, diverse career pathways should be designed, incorporating the strengths of Chinese TCM education to establish a localized acupuncture style education model tailored to the U.S. context.

### 4.2 Enhancing the quality of textbooks and exam content

Textbooks are a critical support for educational activities (43). In the U.S., TCM acupuncture courses largely rely on official English translations as standard texts, whereas materials for acupuncture styles primarily derive from the works of style representatives, lacking uniformity and organization (1, 31). According to structuralist pedagogy, quality learning materials are pivotal in engaging students. Thus, developing international textbooks requires preserving the completeness of TCM theories while employing flexible translation strategies and refining content structure to meet the needs of foreign students. Textbooks for style education should integrate modern research findings and classical TCM content, while also encouraging participation from representatives of acupuncture styles to ensure the scientific and practical relevance of the material. On one hand, the materials should showcase the characteristics of the styles, and on the other, provide insights for integrating styles with mainstream TCM acupuncture. Furthermore, the NCCA certification exam should revise its theoretical portion in alignment with improved textbooks, elevating the weight of TCM theories and classical material while integrating findings from contemporary acupuncture research. The practical exam should also raise its standards by including case analysis and acupoint prescription design in the assessment scope to enhance students’ clinical practice skills.

### 4.3 Enhancing theoretical instruction and advancing comprehensive practice

Experimental research and classical interpretations are vital methods for developing acupuncture theory. As branches of the acupuncture discipline, various acupuncture styles share the common goal of optimizing treatment plans (1, 7, 26). Practical aspects of acupuncture education should prioritize providing students with formalized clinical internship settings. By expanding cooperation with domestic TCM universities, schools can establish long-term collaborative mechanisms with affiliated hospitals to offer students more systematic observation and internship opportunities. The integration of theoretical and practical instruction can be enhanced by using online resources (such as digital databases, multimedia materials, and interactive learning platforms), Virtual Reality (VR, immersive simulation environments), and Augmented Reality (AR, overlaying digital information onto the physical environment) technologies to present content and optimize learning outcomes. Additionally, the TCM apprenticeship model should be explored for incorporation into institutional education, providing insights for overseas TCM education. Constructivist teaching theory advocates knowledge construction through social interaction and contextual learning. Thus, a one-on-one mentoring model pairing TCM teachers with students can guide students in applying theoretical knowledge to clinical practice.

### 4.4 Promote the dissemination of TCM culture and enhance international recognition

International attention is primarily on the practical applications of TCM, with limited acknowledgment of its theoretical and cultural depth (9, 44). This has resulted in a widespread “technique over philosophy” approach in acupuncture education abroad. TCM theory is rooted in a rich cultural background, with its core being a systematic explanation of the perspectives on life, the human body, and diagnosis. The dominance of Western medical culture has diminished the prominence of TCM theoretical frameworks in acupuncture education, particularly in the areas of concept comprehension and cultural outreach. Constructivist teaching theory advocates active knowledge construction by learners through experiential learning, critical thinking, and interactive engagement with the material and context (45). Techniques such as “scaffolding,” which provides temporary supportive frameworks tailored to student needs; “anchoring,” which connects new information to students’ pre-existing knowledge; and “random entry,” which allows students multiple entry points to engage with complex subjects, enhance learning by accommodating diverse learning styles and fostering deeper conceptual understanding (46, 47). Therefore, acupuncture style education should focus on adapting TCM theory to Western culture while strengthening the dissemination of TCM culture. Chinese-American acupuncturists are encouraged to advance the synergy between acupuncture education and TCM cultural promotion, embedding TCM cultural aspects into coursework and interweaving cultural education with theoretical and clinical training. Cultivating TCM-oriented thinking in students facilitates the blending of scientific and humanistic cultures, thereby increasing global appreciation for TCM theories and cultural significance. Adopting structuralist pedagogy emphasizes creating structured, standardized curricula and rigorous assessment systems, while constructivist approaches encourage interactive learning environments, where students actively construct knowledge through contextualized clinical experiences.

## 5 Implications of U.S. acupuncture education for international TCM education

### 5.1 Advancing the standardization of TCM education

Standardization serves as a vital mechanism for ensuring quality in the steady progress of international TCM education and preserving the theoretical framework’s integrity in its global outreach (48, 49). The IIME in 2020 introduced the “Global Minimum Essential Requirements in Medical Education,” encompassing basic medical science and clinical skills while outlining five fundamental competencies: professional ethics, communication, public health, healthcare systems, information management, and critical thinking. Recently, the significance of these five professional competencies has grown in TCM’s global expansion (50, 51). Establishing international TCM assessment criteria requires addressing both theoretical and clinical basics while accommodating the specific context of medical education and TCM development in different nations. This process involves tailoring curricula to local licensure standards, assessing the capabilities needed for independent practice, and crafting adaptable syllabi for effective teaching. Standardizing acupuncture education aims to ensure the systematic and sustainable global development of TCM. The principal objective of advancing international TCM education is to produce qualified practitioners who can deliver clinical TCM services locally, facilitating its incorporation into and improvement of regional healthcare systems. China’s government and related agencies should leverage their authority to foster high-level intergovernmental dialog and strengthen official partnerships internationally. Concurrently, initiatives should promote TCM legislation abroad, forming tiered certification frameworks and quality evaluation systems for TCM education. Develop an assessment framework suited to acupuncture education’s unique traits, driving the standardization and regulation of global TCM education.

### 5.2 Integrate domestic and international educational resources

Promoting multi-level and diverse forms of TCM overseas education is key to improving the internationalization of TCM education. In this process, collaboration and resource sharing between domestic and international institutions should be strengthened, launching quality programs for international students and promoting two-way exchange between faculty and researchers (52, 53). Chinese TCM universities should reinforce internal development, improve teaching quality, broaden partnerships with research institutions and affiliated hospitals, and actively participate in international TCM education and research networks. Meanwhile, universities should be encouraged to offer courses and related majors in international dissemination of TCM culture, cultivating high-level talent focused on TCM cultural promotion. With integrated resources, initiatives like faculty exchange, student exchange programs, and partnerships with recognized foreign universities can be advanced. Within the scope of legal and policy allowances, differentiated talent development plans can be formulated, enabling effective dissemination of TCM theory and culture through mechanisms like degree collaboration and credit transfer recognition (54, 55). Additionally, efforts should focus on high-quality translation of TCM classics and theories, cultivating interdisciplinary talents (proficient in TCM, foreign languages, and communication technology), and forming stable TCM translation and teaching teams to ensure accurate translation of classical works and systematic teaching content. To enhance TCM education approaches, studies can compare the curricula, teaching languages, clinical competencies, and cultural affinity of overseas and domestic TCM students, identifying strengths and weaknesses to propose constructive solutions for TCM education and cultural dissemination globally.

### 5.3 Drawing lessons from international TCM education reforms

The rapid development of international TCM education has raised higher demands for cultivating advanced TCM talent. With the growing influx of international students studying TCM in China, recent years have seen a wealth of teaching reform experiences that offer valuable insights for improving domestic TCM education models. For overseas learners with diverse cultural backgrounds and varying knowledge structures, it is beneficial to transition from traditional, teacher-centered approaches to constructivist pedagogical strategies, emphasizing interactive, problem-oriented learning. Constructivist methods, such as “scaffolding” (initially offering detailed clinical guidance that gradually diminishes as students become proficient), “anchoring” (relating complex acupuncture theories to simpler, familiar medical or cultural concepts), and “random entry” (providing multiple entry points—such as theoretical frameworks, clinical cases, or hands-on practical exercises—for students to explore acupuncture topics), facilitate the creation of interactive and adaptive learning environments, thereby significantly enhancing students’ ability to effectively apply theoretical knowledge in clinical practice (56, 57). Domestic TCM education faces challenges including a gap between theoretical curricula and clinical practice, fragmented theoretical instruction, and the sidelining of core courses. To address these issues, TCM education can draw upon system-focused curriculum reforms from modern medicine and the standardization practices of international TCM education, identifying overlaps to inspire innovative thinking in TCM pedagogy. These efforts aim to support the enhancement of TCM educational quality and its global advancement.

### 5.4 Preserving the scientific and cultural integrity of traditional medicine

Traditional medicine has both scientific practice and cultural tradition attributes. However, the legalization process of acupuncture in the U.S. mainly focuses on its scientific validation, often neglecting its cultural value. The WHO and other institutions advocate for protecting the cultural and societal contexts of traditional medicine during its transmission and dissemination (58, 59). This perspective reminds us that in the overseas development of TCM acupuncture, excessive localization should be avoided to preserve its uniqueness. The historical spread of acupuncture in Japan and Korea illustrates how cultural dilution can integrate the TCM brand into local medical frameworks (10, 11, 23). Avoiding the over-localization of acupuncture requires maintaining its cultural core even while pursuing innovations. For example, American acupuncturists modify tools and techniques to meet market demand, which aids localization but risks diluting traditional culture (60–62). Additionally, efforts should focus on enhancing the authority of independent acupuncture oversight committees. Currently, most states assign the management of acupuncture to biomedical boards, resulting in discriminatory policies against non-physician acupuncturists. While some states have created acupuncture advisory or review boards, their influence remains restricted. To ensure the independence and standardization of acupuncture, the regulatory authority of independent acupuncture boards must be expanded. Moreover, cultural campaigns and advocacy can increase societal accessibility and acceptance of TCM acupuncture. Globally, structuralist theory supports the establishment of standardized, internationally consistent educational frameworks for acupuncture, which ensure clear regulatory structures and maintain the integrity of TCM’s foundational theories. Concurrently, constructivist theory advocates for interactive, culturally responsive teaching methods tailored to diverse learners, promoting active engagement and facilitating deeper comprehension and appreciation of acupuncture’s rich cultural and philosophical dimensions. Integrating these theoretical approaches enables a balanced preservation of acupuncture’s scientific credibility and cultural authenticity, thus enhancing its international acceptance and sustainable development.

## 6 Conclusion

The development of acupuncture education in the United States provides valuable insights into the global dissemination and adaptation of Traditional Chinese Medicine (TCM). Despite significant achievements in integrating diverse acupuncture schools and promoting standardization, challenges such as fragmented curricula, insufficient theoretical grounding, and cultural disconnects persist. Addressing these issues requires a multi-faceted approach: enhancing oversight mechanisms, reforming curricula, standardizing textbooks, and elevating exam content to prioritize TCM’s theoretical depth. Moreover, advancing international collaboration and fostering cultural appreciation are essential for preserving the scientific and cultural integrity of acupuncture. By bridging the gaps between Eastern and Western medical paradigms, U.S. acupuncture education can serve as a model for developing globally recognized systems of TCM education, fostering a balance between cultural heritage and modern innovation to meet the growing demands of global healthcare.
